# Vasectomy provider decision-making balancing autonomy and non-maleficence: qualitative interviews with providers

**DOI:** 10.12688/gatesopenres.15036.2

**Published:** 2024-10-07

**Authors:** Alison T. Hoover, Dominick Shattuck, Karen L. Andes

**Affiliations:** 1 Hubert Department of Global Health, Emory University Rollins School of Public Health, Atlanta, Georgia, 30322, USA; 2 Center for Communication, and Department of Health Behavior and Society at the Bloomberg School of Public Health, Johns Hopkins University, Baltimore, Maryland, 21205, USA; 3 Department of Behavioral and Social Sciences, Brown University, Providence, Rhode Island, 02903, USA

**Keywords:** Vasectomy, sterilization ethics, provider training, male engagement, regret, autonomy, non-maleficence

## Abstract

**Background:**

Male sterilization, or vasectomy, is 99.9% effective at preventing pregnancy with less than a 2% risk of complications. Despite the high efficacy, low risk, low cost, and gender equity benefits of vasectomy, just 2% of women reported that they and their partners relied on vasectomy as their contraceptive method globally in 2019. Health care providers can be both a facilitator and a barrier in men’s health generally, and may be in vasectomy provision as well. This study sought to describe the decision-making rationales of experienced vasectomy providers when evaluating patient candidacy in complex cases.

**Methods:**

Fifteen vasectomy providers belonging to the global Vasectomy Network Google Group from seven countries participated in online interviews using a semi-structured in-depth interview guide. Providers were asked about their vasectomy training, their reasons for vasectomy provision, challenging cases they have faced, and approaches used to manage challenging cases. Vignettes were used to further elicit decision-making rationale. Thematic analysis was conducted using MAXQDA20.

**Results:**

Provider decision-making was predicated on ensuring patients were well-informed, able to consent, and certain about their choice to have a vasectomy. Once those foundational conditions were met, providers filtered patient characteristics through their training, laws and policies, sociocultural norms, experience, and peer influence to produce a cost-benefit breakdown. Based on the cost-benefit analysis, providers determined whether to weigh autonomy or non-maleficence more heavily when determining vasectomy patient candidacy.

**Conclusions:**

Despite clinical best practices that promote prioritizing patient autonomy over non-maleficence, some providers continued to weigh non-maleficence over autonomy in vasectomy patient candidacy evaluations. Non-maleficence was particularly prioritized in cases providers deemed to be at higher risk of regret. The findings of this study suggest vasectomy provider training should emphasize evidence-based best practices in shared decision-making and patient-centered care to facilitate vasectomy provision that honors patient autonomy and rights.

## Introduction

Contraceptives play a key role in meeting individuals’ right to plan if, when, and how many children they will have toward attaining the highest standard of sexual and reproductive health ( United Nations Department of Economic and Social Affairs, 2019b). The global availability has halved fertility rates around the world from 4.7 births per woman in 1960 to 2.3 in 2021 ( The World Bank, n.d.). Sterilizations—both male and female—are the most effective contraceptive methods currently available, and male sterilization, or vasectomy, is the only authorized contraceptive method for men besides condoms. A vasectomy is a safe, simple, and highly effective permanent procedure that involves disrupting the flow of sperm into the seminal fluid by severing each vas deferens through a small opening in the scrotum ( Shih *et al.*, 2014). Vasectomies are 99.9% effective at preventing pregnancy, with less than a 2% risk of complications, including infections, hematomas, or chronic pain ( Shih *et al.*, 2014). Beyond the clinical benefits of vasectomies, studies have found an association between men who utilize vasectomy and report willingness to use novel male contraceptives and more equitable gender norms ( Nguyen & Jacobsohn, 2023; Nicholas *et al.*, 2021; Stern *et al.*, 2015).

Despite the high efficacy, low risk, and low cost of vasectomy, globally just 2% of women reported that they and their partners relied on vasectomy as their contraceptive method in 2019, making it one of the least-used forms of contraception ( United Nations Department of Economic and Social Affairs, 2019a). The unmet potential for vasectomy uptake is particularly clear when comparing directly to female sterilization (tubal ligation), which is 20 times more likely to have major complications, 10 to 37 times more likely to fail, and on average costs three times more than vasectomies ( Hendrix *et al.*, 1999). Despite this, female sterilization is the most common contraceptive method worldwide, accounting for 24% of contraceptive use (219 million women). Between 1994 and 2019, global vasectomy use decreased from 3% to 0.8%. ( United Nations Department of Economic and Social Affairs, 2019a).

The contributing factors to low global vasectomy uptake are multifactorial and interrelated, but are best understood as policy, demand, and supply barriers. Policy barriers that include limited public funding and mandatory waiting periods undermine health infrastructure and routine access to the method. Persistent low demand for vasectomy that is fueled by lack of awareness, myths and misconceptions, and gendered norms surrounding virility and male health seeking behaviors additionally contribute to limited uptake ( Shattuck *et al.*, 2016; Shelton & Jacobstein, 2016; Shih *et al.*, 2013; Shih *et al*., 2014). Supply barriers to vasectomy uptake include vasectomy provider-imposed eligibility limitations, lack of knowledge among general non-vasectomy providers, bias among reproductive health providers in working with men, and low vasectomy provider availability.

Notably, the evidence is sparse on supply barriers for vasectomies. Although it has been suggested that the supply is not the issue ( Shelton & Jacobstein, 2016), concerns have been raised about biases and knowledge among non-vasectomy providers. A recent systematic review of barriers to male participation in general reproductive health care highlighted that barriers to inclusive and integrated quality reproductive health services are the primary limitation to male engagement in reproductive health, and pointed to health care workers as a major contributor to the lack of access to reproductive health services ( Roudsari *et al.*, 2023). Another recent study similarly highlighted that many health professionals involved in contraception have unsatisfactory knowledge of male contraceptive methods and are not reliably offering male options during couple contraceptive counseling sessions ( Tcherdukian *et al.*, 2022). Measuring the impact of provider bias poses major methodological challenges, but several studies have shown that provider bias has a significant impact on provider contraceptive provision practices and may lead to reduced contraceptive access ( Solo & Festin, 2019). Documented biases include but are not limited to: not providing contraceptives to young people, pushing highly effective contraceptive methods instead of a patient’s stated preferred method, not counseling individuals without children on contraception, and omitting side effects from contraceptive methods during counseling ( Bullington *et al.*, 2023; Solo & Festin, 2019).

Nearly all the research that has been undertaken about reproductive health provider bias has focused on women and women-controlled contraceptive methods. There is a need to explore how vasectomy providers evaluate patient candidacy, and their role in facilitating or limiting access to reproductive health services like vasectomy, to design appropriate interventions to support vasectomy uptake at the provider level. The purpose of this study was to describe the decision-making rationales of experienced vasectomy providers that belong to the global Vasectomy Network Google Group when evaluating patient candidacy in complex vasectomy cases. The findings can be used to design trainings and policies that address supply gaps in vasectomy access.

## Methods

### Study design & participant recruitment

This study used a descriptive cross-sectional qualitative design using in-depth interviews (15) with vasectomy providers to describe their decision-making rationales for vasectomy service provision. Participants were recruited through the Vasectomy Network Google Group (VNGG), which, at the time of recruitment, was comprised of 535 providers from more than 30 countries. Authors AH and DS were members of the VNGG through prior work. Inclusion in this study was limited to current membership in the VNGG and providing vasectomy services for a minimum of 5 years. The criteria ensured a breadth of experience with different case contexts that would inform their decision-making process. These criteria also ensured the participants were drawing on actual experience and not hypothetical decision-making, known to be subject to bias.

An email was sent to all participating VNGG members describing the study and its objectives and inviting those interested to fill out an online screening form. The screening form queried their name, email, primary country of vasectomy provision, age, number of years continuously providing vasectomies, and availability for an interview. In total, 22 providers completed the screener, and one provider completed the screener twice for 23 total responses. Of the 22 that completed the screener, 20 were eligible; two providers had not provided vasectomies for five or more continuous years. Of the 20, 15 were able to participate in an interview during the subsequent month. After the 15 interviews took place, the study team determined saturation had been reached and no further interviews were scheduled.

### Ethical considerations

The study was approved by the Emory University Institutional Review Board (Approval #00001730, issued November 19, 2020). Verbal consent was taken from all participants. The Emory IRB approved a waiver of written consent given: 1) the research presented no more than minimal risk; 2) involved no procedures for which written consent is normally required outside the research context; 3) the interviews were conducted over zoom, and written consent would have required e-signatures or printing, signing, scanning, and emailing consents, deemed to pose a burden to a group of infrequent technology users. The Emory IRB determined that recording verbal confirmation of participant consent minimized burden while ensuring full consent. All interviews begin with a review of the consent form, which was sent to participants ahead of time, and recorded verbal consent. 

### Data collection

Interviews were conducted by female author AH using Zoom video calls and lasted approximately one hour. At the outset of interviews, the interviewer went through the consent form and sought verbal consent. All interviews were audio and video recorded, with participants’ consent.

Data were collected through semi-structured in-depth interviews to describe decision-making rationales of vasectomy providers ( Hoover & Andes, 2024). The interview guide began with close-ended questions to capture key demographic information, including age, gender, country performing vasectomies, years providing vasectomies and training background. Open-ended questions then followed about their training in vasectomy provision, their reasons for getting involved in vasectomy provision, challenging cases they have faced in their career, and the approaches they use to handle such challenging cases. Vignettes were used to help further elicit decision-making rationale. Vignettes included the following case studies: 24-year-old single childless male motivated by overpopulation and climate change; 30-year-old male with a currently pregnant partner; 50-year-old recently divorced male; 37-year-old male expressing visible discomfort about the idea of the procedure; and 34-year-old male who does not disclose any disorders but lists lithium on his medications list.

Interviews were conducted principally in English, with one interview conducted in Spanish. An IRB-approved Spanish consent form was used with this participant.

### Data analysis

 All interviews were transcribed verbatim. The Spanish transcript was transcribed in Spanish and then translated into English for analysis. Transcripts were then uploaded into MAXQDA20, a software package for qualitative data analysis (VERBI GMBH, Berlin). Data analysis involved reading transcripts multiple times and memoing data to identify core themes, which were then developed into a codebook with both inductive and deductive themes. The research team, comprised of two individuals, iterated the codebook, the final version of which was used by author AH to code all 15 transcripts.

 Thematic analysis involved drafting detailed summaries for each code, examining properties, dimensions, and variation across and within participants. This resulted in a rich description of the factors involved in provider decision-making, as detailed below. Results were compared across age, gender, country, medical specialty, years providing vasectomies, and average number of vasectomies performed each month. No clear distinctions were uncovered among these characteristics. Most of the providers factored similar considerations into their ultimate decision-making, which is described in aggregate in the results. The full transcripts were frequently reviewed to verify and contextualize results.

## Results

In total, 15 providers were interviewed, ranging in age from 41–71 years (see Table 1 for descriptive statistics). Four were urologists, five were general practitioners, and six were family medicine doctors. As there was not a meaningful distinction between family medicine providers and general practitioners in their training, providers will be presented as urologists and non-urologists for the purpose of this analysis.

**Table 1.** Demographic characteristics.

	Overall (n=15)
**Gender**	
Female	1 (6.7%)
Male	14 (93.3%)
**Age**	
Median [Min, Max]	65 [41, 71]
**Years providing vasectomies**	
Median [Min, Max]	30 [6, 43]
**Average number of vasectomies performed each month**	
Median [Min, Max]	27 [10, 275]
**Medical specialization**	
Family Medicine	6 (40.0%)
General Practitioner	5 (33.3%)
Urology	4 (26.7%)
**Country where they perform the majority of vasectomies**	
Australia	1 (6.7%)
Canada	1 (6.7%)
Ireland	1 (6.7%)
Mexico	1 (6.7%)
Spain	1 (6.7%)
UK	3 (20.0%)
USA	7 (46.7%)

**Figure 1.**
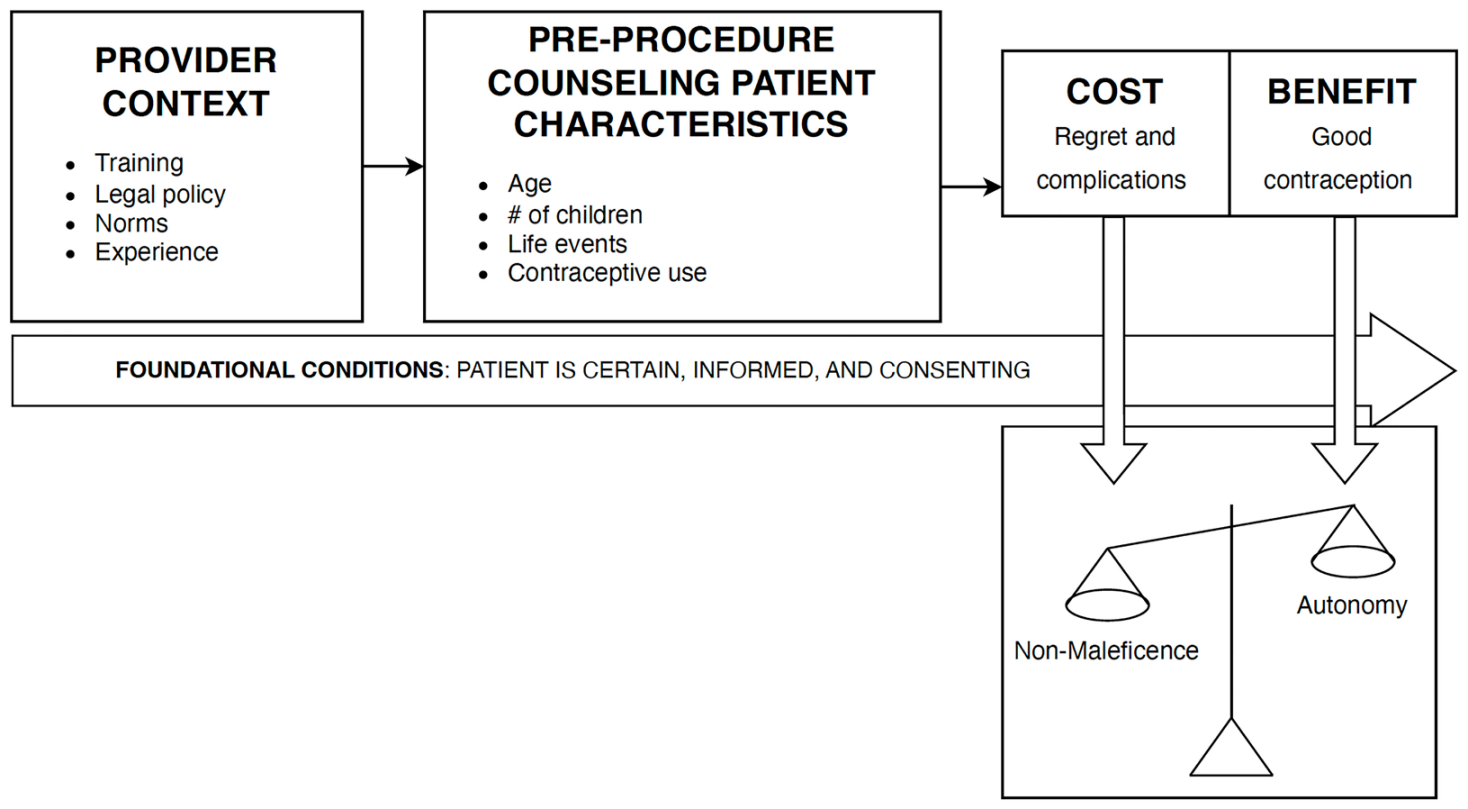
Pre-procedure decision-making flow.

Three main factors were identified as foundational to vasectomy provider decision-making: 1) patients’ knowledge about the procedure, 2) patients’ clear understanding of consent prior to the procedure, and 3) patients’ clarity in choice to have a vasectomy. Once those conditions were met, providers filtered patient demographics through their training, local laws and policies, and experience in order to evaluate the cost-benefit breakdown for particular patients. The cost of potential regret or complications may outweigh the benefit of desired contraception, or vice versa. Based on that cost-benefit analysis, providers then determined whether or not they weighed autonomy or non-maleficence more heavily during the pre-procedure decision-making process ( Figure 1). 

### Role of pre-procedure counseling

*Informed consent is a process…about finding out who the patient is so that you know what they need to know. – Participant 2*

Pre-procedure counseling discussions were the primary stage and setting where providers determined and decided patient candidacy for a vasectomy. Counseling discussions offered an opportunity for providers to educate and for patients to provide their context, with the final decision from the provider often being provided by the end of the counseling session. The stages of the counseling discussion lay the groundwork for the decision-making flow, and consist of a thorough overview of the procedure and technique, how vasectomy prevents pregnancy, risks, and recovery and care instructions. Providers also emphasized the need for clients to understand that the vasectomy procedure should be viewed as permanent, given variable reversal success rates. All providers described reviewing other non-permanent contraceptive alternatives, and some providers discussed sperm storage options with patients if available, particularly for younger patients. Providers often reviewed medical charts before or during the counseling session, providing an opportunity to solicit additional information deemed essential to candidacy decision-making.

All providers’ pre-counseling processes included discussions of the risks of the procedure, though the extent to which the risks were discussed varied. In addition to the direct physical risks of the procedure, most providers counseled on possible indirect outcomes of the procedure, particularly those that might lead to vasectomy regret, such as separating from the current partner, losing a child, or simply changing one’s mind. Some described this as “putting [the decision] in perspective,” or “checking in,” and many considered this a critical step in avoiding patient regret. For example, one provider asks patients with pregnant partners if they would be ok if they could not have any more children and they lost this pregnancy in an unlikely obstetric disaster. From there, providers answer questions; some providers ask patients to repeat the process back to them to verify comprehension, and then providers document consent.

*My responsibility is to try to put things in perspective in case [the patient] has not put them in perspective, because of the permanent nature of this decision. – Participant 14*

Pre-procedure counseling was considered valuable for protecting patients from vasectomy regret or coerced procedures; one provider believes the practice of pre-procedure counseling is part of overcoming the legacy of forced sterilization. Some providers also highlighted the importance of pre-procedure counseling as malpractice lawsuit protection for medicolegal cases. A few providers considered the process of documenting consent to be just as important as the consent itself.

 Providers’ pre-procedure counseling approaches and steps were informed by their peers, local laws and guidelines, and their experiences. Some providers referenced consent checklists they use that came from the VNGG, and another said their consent process has improved over the years as they have gone about accrediting other providers and seeing their approaches. Several providers reflected that their approach to pre-procedure counseling is a transference of their other medical training. One provider uses the direct language set about in the American Urological Association guidelines, while all UK providers have set language and standards they must adhere to under their medical board, in part informed by a historic legal case, *Montgomery* v *Lanarkshire Health Board*, regarding informed consent. Experiences of being sued, having complaints levied by the medical board, or serving as an expert witness in medicolegal cases also informed some providers’ approaches to the consent process. Two providers who offer reversals noted that seeing who comes back for reversals has shaped how they counsel vasectomy patients with overlapping characteristics. It is worth noting that in a legal review of US vasectomy litigation, there were 67 vasectomy-related cases from 1990–2017, 29.9% of which were for negligence of informed consent. Of the negligence in informed consent cases, most were a result of a wrongful pregnancy and 90% were decided in favor of the defendant physician ( Blazek *et al.*, 2019).

### Foundational characteristics: fully informed, able to consent, & certainty

*Now, if they if they feel it's the best thing for them, and they've convinced me that they've thought about what they're doing, I'll move ahead. – Participant 15*

Pre-procedure counseling validated three foundational and essential components of patient candidacy: fully informed, able to consent, and certainty. The conversational nature enabled providers to discern patient mental status and ability to consent, to ensure patients understood what they have been told and were fully informed. Providers also used the encounter to evaluate patient rationale and certainty about their decision, while managing patient expectations of the procedure and the possibility of regret. If providers deemed the patient to be unable to consent, uninformed, or uncertain, they would not proceed with offering the procedure.

Patients who were not fully informed and would not be candidates for a vasectomy would include those seeking a vasectomy because they think it will resolve their erectile dysfunction or premature ejaculation, those who are convinced they will have erectile dysfunction after the procedure, or those who did not listen during counseling. Providers described that once informed, patients needed to give consent free from coercion, duress, grief, major life changes, manic or depressive episodes, hesitation, or uncertainty. Providers described the need for patients to be mature and capable of giving consent through their physical and mental age, in line with local age of maturity laws. Providers described using additional counseling for patients with mental health conditions. No providers said that mental health conditions automatically excluded clients from vasectomy eligibility, but all said they would take measures to ensure the condition was well-controlled without recent depressive or manic episodes, and the patient was able to give fully informed and valid consent. Some providers were comfortable proceeding in instances where patients could not give fully informed and valid consent, as long as they had legally valid consent from a parent or guardian or a patient’s primary care provider or psychologist. For example, in the case of an individual with an intellectual disability who was dependent on care takers, some providers would provide a vasectomy after consultation with the individual’s legal guardian and a secondary medical opinion, such as from a psychologist. Providers also screened out patients who might be selecting vasectomy as a form of retaliation against an intimate partner, or as a form of self-harm.

 Most providers also sought a sense of certainty from all patients about their decision to pursue a vasectomy. A few providers described this phase of consent as asking patients to “convince” the providers of their decision. Patients that were considered certain about their decision were described as adamant and insisting on the procedure, while those who were uncertain asked repeatedly about sperm banking or the success rate of vasectomy reversals. Other characteristics that factored into perceived certainty included the time a patient spent considering a vasectomy, their motives and rationale for wanting a procedure, and their thoughtfulness on their potential to regret the decision in the future. The appropriate amount of time spent considering a vasectomy depended across the providers, but most providers wanted a minimum of one month considering the procedure. Providers suggested that older patients did not need to consider the decision as long as younger patients to be considered appropriately thoughtful about the decision. Many providers also asked patients directly, particularly young patients, about whether they had considered the potential of regretting the decision at a later point and if they had considered other contraceptive alternatives for the time being. Patients that exhibited complex decision making, or acknowledged the potential for regret but still wanted the vasectomy, were more likely to be considered certain and therefore candidates for vasectomy than those who did not acknowledge the potential for regret.

### Patient characteristics

Pre-procedure counseling was also an opportunity to look for signs of elevated risk of regret based on patient characteristics and life stage. Providers factored in age, number of living children, marital status, job and insurance status, current contraceptive use, partner’s pregnancy status, mental health conditions, family history, and fertility in conjunction with one another to determine the level of counseling needed and as part of decision-making. Age was the biggest predictor of additional counseling, followed by number of children, pregnancy status, and presence of psychiatric conditions.

Age: Younger and older patients were more likely to get additional counseling and potentially have their procedure delayed or denied. Younger patients were commonly considered to have the highest potential for regret, which led to providers doing additional counseling on regret as well as examining other patient characteristics to determine vasectomy eligibility. Other patient characteristics that were more heavily examined with a young patient were number of living children, relationship or marital status, contraceptive alternatives, stance on abortion, and health history. The definition of “young” varied by provider, with some citing 21 as the age of full adulthood, others using what they called an arbitrary 27, and others considering anyone under 30 to be young and meriting additional counseling.

*…like all of us, I would have a strong and detailed discussion with a young man who's requesting a vasectomy, emphasizing the fact that reversals are expensive, and don't always work. – Participant 8*

Older patients and those with older partners at or near menopause also received additional counseling. Some providers detailed that only two or three years of contraceptive protection did not seem worth the risks of complications from a vasectomy procedure and would encourage their patients to consider other contraceptive methods until their partner had begun menopause. This rationale did not apply in instances where the patient was single or not in a committed partnership with someone near menopause, such as if an older man was with a younger partner.

Number of children: Patients with no children or one child received more counseling than patients with two or more children. Some providers sought out a psychologist to verify the patients’ ability to give consent and make informed decisions if they did not have children. Providers also described examining the relationship or marital status of patients without children alongside contraceptive alternatives; one provider acknowledged the realities and demands of modern parenting and perceived ability to be a father in their decision-making.

Pregnancy: For patients whose partner was currently pregnant, some providers said they would be sure to provide additional counseling about the risks of fetal or newborn demise. This also depended on how many living children the patient had, and whether this pregnancy was intended.

Marital status: Only one provider noted factoring marital or relationship status on its own into their decision-making on vasectomy eligibility. This provider was wary of providing vasectomies during or after any major life changes, such as a divorce. Others described using this indicator only when other characteristics may have raised questions about vasectomy eligibility.

Contraceptive alternatives: A few providers examined contraceptive alternatives if there were lingering questions about patients without children or who were on either the younger or the older end of the age spectrum. This entailed whether there were viable contraceptive alternatives for the couple to use that are reversible or had a smaller risk of complications, such as an IUD. For one provider, this also included considering the couple’s stance on abortion and whether methods with a higher failure rate would be deemed unacceptable. 

Family health history: A couple of providers described instances of family history as factoring into a patient’s candidacy. One provider had a patient with an incurable hereditary disease he did not want to pass on to his children, so though he was young and childless, this fact increased his eligibility for a vasectomy. Another provider had a young and childless patient with a family history of addiction and his own patterns of drug use, and the provider intended to move ahead with offering the procedure.

Other: Less commonly, some providers factored fertility and employment and insurance status into decision-making. A couple of providers noted that they did not consider men who reported using testosterone as candidates for vasectomy without a semen analysis indicating high levels of sperm concentrations, given sterility is a common side effect of testosterone use. One provider weighed employment status; patients with stable employment were less likely to be making an impulsive decision about vasectomy and were considered better candidates for vasectomy than those with unstable employment. And one provider allowed that he would be more likely to provide the vasectomy if the patient’s insurance would soon be expiring and they had a short window in which to pursue sterilization or contraceptive methods in general.

### Provider landscape

The combination of patient characteristics was filtered through the provider’s landscape of training, experience, norms, laws and guidelines, and peer influence as part of determining the cost-benefit breakdown of a vasectomy for a given patient.

Training: Provider training in the vasectomy technique itself was described as difficult to acquire. Of the 15, four were urologists and 11 were non-urologists. All the urologists received training on traditional vasectomy techniques during their residency, but only two of the family medicine doctors received their initial training on vasectomy during residency. The rest learned the technique through private or on-the-job training. One family medicine provider noted that it is becoming increasingly uncommon for family medicine providers to be trained on vasectomy, and the reduction in vasectomy training availability for family medicine practitioners was echoed by two other providers who described facing push-back and significant challenges to securing training.

Only three providers (20%) reported receiving formal and vasectomy-specific counseling training. Even providers who received vasectomy training during residency reported learning how to conduct pre-counseling and document consent through their general medical training and had not received vasectomy-specific counseling training. One provider expressed an explicit desire for medical ethics training as it related to vasectomy. Overall, providers in this sample had not received guidance or training on male sterilization-specific pre-procedure counseling, turning instead to experience, peers, and national regulatory guidelines. 

Experience: Most providers highlighted that accruing experience had improved their approach to counseling and made it more comprehensive. Reasons providers adjusted their counseling style ranged from malpractice suits, joining a new surgical practice, and exposure to other methods of counseling and consent. One provider serves as an expert witness in medicolegal cases and noted that exposure to those cases significantly affected the way they practice including how they collect consent and how they document consent. While most providers felt that experience had led to improved decision-making, two mentioned becoming more conservative as they managed ethically challenging cases. One noted, “experienced surgeons step back from more procedures than inexperienced surgeons,” and having seen a young patient come back and seek a reversal, he had grown more cautious with decision-making.

Peer Influence: Many of the providers described calling on peers to consult on challenging cases, improve upon their respective pre-counseling and consent protocols, and seek feedback on retrospective clinical and sociocultural approaches. Providers gave examples of a variety of peers, including psychiatrists, general practitioners, clinical assistants, online networks, medical missions, colleagues in their physical office, doctors they met at conferences, and doctors met through reaccreditation processes. In challenging cases including young men or those with intellectual disabilities or mental health conditions, most providers mentioned they would seek out a second opinion. Providers’ reasons for soliciting a second opinion included to build their confidence with their approach as well as for legal coverage in the future if their decision-making was drawn into question. The providers generally felt that having a second provider evaluate a patient supported more robust decision-making.

Sociocultural Norms: It was somewhat uncommon for providers to highlight sociocultural norms as influential in their decision-making. Of those that did acknowledge the influence of sociocultural norms, they focused on social expectations of having children, political climates, and health justice issues. All providers who highlighted the social expectation of having children as influencing a patient’s candidacy for vasectomy were US providers. They noted a potential double standard in themselves and in other providers that they felt a greater need to prevent regret around not having a child than they felt the need to prevent regret for having a child. This included age double standards – it was generally considered ok among providers for a 24-year-old to have kids but not for a 24-year-old to decide to get a vasectomy.

*By saying no to him [denying a vasectomy], I'm forcing, in some ways, forcing him to be a father. Just because in our society or in ourselves, we feel bad for people if they accidentally miss out on the chance of becoming a parent. – Participant 1*

Providers also acknowledged their decision-making was influenced by the social attitudes of the countries in which they practice. One non-US provider practices in a conservative country that has been slow to implement a vasectomy program. This participant noted that the general resistance to vasectomy and male involvement in family planning resulted in his implementing highly cautious procedures. A major concern for this provider is to avoid poor outcomes (such as regret) that could impact the promotion of vasectomy more widely. Conversely, providers in two less conservative countries noted social attitudes about the importance of patient autonomy influenced their approach to decision-making.

Laws and Guidelines: Providers described laws and guidelines in each country that governed access, rights, and pre-procedure protocols. Many of the countries represented in this study have a history with forced or coerced sterilization, where vasectomies were conducted without the knowledge or against the wishes of the patient for reasons including but not limited to population control and eugenics ( Sheynkin, 2009). Among the locations present in this study, the UK was the only country that required a referral to see a vasectomy surgeon. Spain was unique in that only surgeons, urologists, or gynecologists could provide vasectomies; the vast majority were provided by urologists. Some providers noted this history influenced their consent process, including through regulatory outcomes of historical legal adjudication.

Providers in other countries mentioned several laws that influenced their protocols. In Australia the Anti-Age Discrimination Act outlaws discrimination against youth and the elderly, which made the Australian provider less likely to turn away younger or older men seeking vasectomy because of their age. In Mexico, the Official Standards for Family Planning decrees the right of an individual to decide when and how many children they want, which the Mexican provider noted makes him more likely to provide a vasectomy on a childless man, even with a chance the man may come to regret it. In the UK, the Mental Health Act confers the ability to suspend personal liberties to a psychiatrist and independent assessor, which influenced the UK providers’ consent process and approach to patients who may not be able to give full and informed consent on their own.

There were also regulations around how to collect consent and who can give consent. In the US, these regulations for how to collect consent came from the American Urological Association guidelines and in the UK, the General Medical Council and the Faculty of Sexual and Reproductive Health Guidelines. The US and the UK also have guidelines and regulations around mandatory waiting periods – the UK has a universal two-week waiting period between the pre-procedure counseling and the procedure. Any procedure that is federally funded in the US, through either Title X or Medicaid, is also subject to a 30-day waiting period between counseling and procedure. The state of New York has the same waiting period for privately funded vasectomies. Providers described numerous ways in which laws and guidelines influenced their practice. One provider in the US noted that because of the 30-day waiting requirement for Medicaid, they expect all their patients to have thought about the procedure for a month before feeling comfortable providing it. For that provider, the 30-day waiting period set a subconscious precedent even for those who are not subject to that regulation. Similarly, because the Medicaid age of consent is 21, another US provider used that as their age of consent for all vasectomies no matter how they are funded.

*Now, what's magic about 21? There's nothing magic about 21, whether it comes to drinking or smoking or anything else. But the fact is that in order to get assistance through the United States government under either Medicaid or Title X, you have to be 21. So I'm just following sort of a guideline that is there. It's very random. – Participant 4*

### Cost – benefit analysis & ethical obligations

Once the foundational conditions of certainty and informed consent were met, providers filtered patient characteristics through their training, laws and regulations, sociocultural norms, experience, and peer influence in order to evaluate the benefits of proceeding with the vasectomy against the costs. Based on that cost-benefit analysis, providers then determined whether to weigh ethical principles of autonomy or non-maleficence more heavily during the pre-procedure decision-making process. Autonomy (respect for the patient’s right to self-determination) and non-maleficence (do no harm) are two of the four principles of medical ethics, alongside beneficence (do good) and justice (treat all people equally and equitably) ( Varkey, 2021). The use of the terms autonomy, non-maleficence, and cost-benefit analysis arose during analysis to capture themes described during interviews. While the terms were used by some providers during interviews, these are primarily terms assigned by the study team.

While all providers felt recognizing patient autonomy was a pillar of service provision, some weighed the principle of non-maleficence more heavily than the principle of autonomy in particular circumstances based on the provider’s evaluation of harm, as determined through their provider landscape. Specifically, a few providers felt that vasectomy regret was a substantial harm, and as such, patients with a high chance of regret were not good candidates for vasectomy. Patients considered to have a high chance of regret included young patients, patients without children, and patients undergoing major life changes including pregnancy or divorce. One provider explained the need to avoid patient regret as predicated on the high cost, low availability, and limited success rate of vasectomy reversal procedures, especially given the availability of well-functioning forms of non-permanent contraceptives. In that cost-benefit analysis, the provider deemed the cost of vasectomy regret to be greater than the benefit of vasectomy as compared to other contraceptive options.

*You want to minimize regret. And you want to make sure people are in the frame of mind when they've thought about all the scenarios, where, if their life changed, that they could consider having all the options available. Not everyone can afford, you know, $15,000 for a reversal, or $20-25,000, for a sperm retrieval and IVF. So you want to make sure that you don't have them burn any bridges. It's like, measure twice, cut once, you know? – Participant 15*

Two providers highlighted the non-zero risks of complications during vasectomies and the potential for physical harm compared to the elective, non-essential need for a vasectomy. These providers noted the low but present risk of infections, hematomas, and chronic pain, and felt that because a vasectomy is an elective, non-essential procedure, that the threshold where risk outweighs benefit should be low. As such, these providers did not consider patients with elevated chances of vasectomy regret or those with low benefits (e.g. men with partners near menopause) to be good vasectomy candidates. They saw themselves as shared decision partners in the process, guiding patients toward reasonable decisions and operationalizing the “do no harm” principle.

*My responsibility is helping them to make the best decision. Most of the time, I'm not making the decision. But in some cases, I'm inclined to orient the patient... I think it's my responsibility to do some shared decision making on what is the best option. – Participant 9*

The third provider that emphasized non-maleficence in decision-making was driven largely by sociocultural norms and the nascency of the vasectomy program. Given the desire to maintain a good reputation and not challenge the legitimacy of the relatively new national vasectomy program, this provider was highly reluctant to take on complicated cases that could result in patient regret or complaints. This provider was acutely concerned with individuals making decisions under duress, including during major life changes such as a pregnancy or a divorce. The provider’s only instance of regret came from a young patient, and the provider’s experience with that case informed their preference to impose waiting periods for young patients.

There were also two providers that equally weighed concerns of non-maleficence against autonomy. The two providers had inclinations to delay patients who were young, childless, or during major life changes but would provide the vasectomy without a waiting period for those who were “insistent” or “adamant” or who had concrete rationales for needing the vasectomy now, such as expiring insurance coverage. The providers were similarly informed by experience, laws and regulations, data around patient regret, and their sense of responsibility as providers.

*I’m a doctor, not a technician…Just because someone wants something doesn’t mean it’s the best thing for them. And it’s my job to lay that out for them. Now, if they feel it’s the best thing for them, and they’ve convinced me that they’ve thought about what they’re doing, I’ll move ahead. But if I’m not convinced, I’m not obligated to do something that I think is the wrong thing for the patient. – Participant 15*

Two thirds of providers factored non-maleficence into their counseling but not into their judgment of vasectomy candidacy. These providers described viewing their responsibility as needing to educate patients and probe about possible life changes and outcomes that could lead to regret, but felt that once patients were thoroughly counseled and able to demonstrate being fully informed, they would provide the procedure. For these providers, patient autonomy superseded their own concerns around regret. Honoring their autonomy included providing a vasectomy even if the provider felt the decision was wrong or had a high chance of regret. This group of providers was informed by experience, training, laws and regulations, and their sense of responsibility as providers.

*…with the underlying belief being that his autonomy, his ability to make his own decisions about his own reproductive destiny, should trump our concerns unless there's something very much impairing his ability to make those choices. – Participant 3*

Ultimately, provider decision-making was multifaceted and reflected the contexts in which they work and how they were trained, in addition to more personal elements of their perceived responsibility as providers.

## Conclusions

Ultimately, vasectomy providers employed contextual factors, lived experiences, and personal values as part of prioritizing the principle of autonomy or the principle of non-maleficence in decision making, particularly in complex cases. Once the foundational conditions of patient certainty and fully informed patient consent were met, providers filtered patient characteristics through their training, experiences, laws and regulations, norms, and personal responsibility. The result was a cost-benefit analysis for the individual patient, where the provider weighed the risk of regret and complications against the benefit of good contraception. This cost-benefit analysis then informed whether providers weighed the principle of autonomy or non-maleficence more highly in decision-making.

 Based on the outcomes of this decision-making process, providers perform, delay, or deny procedures. Prioritizing autonomy commonly meant doing the procedure and emphasizing non-maleficence most often meant delaying or denying the procedure. For example, a young, unmarried, and childless patient seeking a vasectomy was deemed by most providers as being at a high risk of vasectomy regret. Providers that prioritized autonomy after the cost-benefit analysis would proceed with the vasectomy. Providers that prioritized non-maleficence would either delay the procedure and impose a waiting period to make sure the patient was sure and had considered the chance of regret or deny vasectomy candidacy until the patient was older.

Many of the providers in this study prioritized autonomy. Some shifted their prioritization on a case-by-case basis, and a few predominantly prioritized non-maleficence. There were no clear patterns based on medical specialization, country of operation, years providing vasectomy, number of vasectomies provided per month or if they also provide reversals that predicted whether a provider would prioritize autonomy or non-maleficence.

 Providers described different assumptions about the role of doctors in medical decision-making processes, which shaped their ultimate prioritization of autonomy or non-maleficence. Within this study, there were two distinct schools of thought about the role of doctors. The first group viewed doctors – and thus, themselves – as educators and a resource available to facilitate patient decision-making. The second group saw themselves as active and engaged arbiter of decision-making. These two operating paradigms are both appropriate and permissible within medical decision-making, but they do have distinct implications for patient care. This analysis will not attempt to evaluate the defensibility of these two paradigms, but rather evaluate their implications.

 The first group, which will be referred to as the “educators,” generally prioritize autonomy over non-maleficence. They saw their role as educating, empowering, and facilitating the patient’s own ultimate decision. The educators held themselves at a distance in decision-making, describing their responsibility as providing information and offering their perspective. These providers felt their responsibility extended as far as providing full and complete information but no further. This was true even if the provider felt that the patient was making a mistake with their decision. One provider offered a metaphor of seeing a patient running toward a cliff, and the provider viewing it as their responsibility to shout, “there is a cliff up ahead!” but not to stop the person if they knowingly continue to run toward the cliff. Educators frequently commented that individuals have a right to make wrong or bad decisions. Educators also more commonly mentioned and considered the full landscape of contraception, including the impact on female partners if the male partner did not have access to a vasectomy.

 The educator approach is strong in the way it honors patient autonomy and promotes patient-centered decision-making. It also limits opportunities for provider bias in determining vasectomy eligibility when the decision is led by the patient. A weakness of this approach is that access to vasectomy reversal services can be limited and costly, and may create differential access to reversal services among those who can afford to pay out of pocket, though this may reflect more structural global health care inequity than the weakness of the approach fundamentally. It may undermine vasectomy programs as word-of-mouth from unsatisfied clients could influence uptake. 

 The second group, which will be referred to as the “arbiters,” generally prioritize non-maleficence over autonomy, and saw their role as extending beyond education. This group viewed the decision as one made in partnership, but where the provider had the ultimate say given the provider’s distance from the emotion of the decision and their familiarity with vasectomy regret. The arbiters tended to emphasize the risks associated with vasectomy, including the risk of regret as well as the risk of rare complications including infection, hematoma, or post-vasectomy chronic pain. Considering these risks, arbiter providers were more cautious and conservative with their approaches. Some arbiter providers described their lead role in decision-making as holding themselves to a higher standard as providers. Most arbiter providers are from socially conservative countries. 

The arbiter approach is strong in that it avoids complications in patients that may come to regret the procedure, and limits vasectomies that result in regret where reversals are either not accessible or not affordable. As such, this approach may reduce costs and overall utilization of health care resources among patients seeking vasectomy. However, it may increase overall utilization of health care resources when taking into account the possibility of unwanted conception or for female partners to resume the burden of contraception. The arbiter approach may reduce the overall incidence of vasectomy regret, though vasectomy regret is rare ( Charles *et al.*, 2023). One provider also highlighted the role of cautious vasectomy provision as part of overcoming the legacy of forced sterilization and the importance of exceptional diligence in decision-making in light of this legacy.

A weakness of this approach is the opportunity to introduce provider bias in determining whether the risk of regret is too high. Recent research suggests childless men are no more likely to regret vasectomy and should not be counseled any differently than men with children ( Bryk *et al.*, 2020; Najari *et al.*, 2021). Denying access to sterilization over the possibility of future regret has also been deemed unethical ( Lalonde, 2018; McQueen, 2017; Mertes, 2017). Yet, childless men were considered at high risk of regret among both educator and arbiter providers, underscoring the potential for bias to shape vasectomy access when arbiter providers take an active role in decision making.

### Current clinical best practices

Shared decision-making is considered a best practice in clinical decision-making and patient-centered care. Shared decision-making entails detailed information on the benefits and harms of the procedure provided by a health care professional as part of facilitating patients to arrive at informed preferences ( Charles *et al.*, 1997; Elwyn *et al.*, 2016). These informed preferences are then respected and integrated into decision-making as a way of respecting autonomy. Shared decision-making in contraceptive choice has been shown to increase satisfaction with both provider counseling and ultimate method uptake ( Dehlendorf *et al.*, 2017). Patients who engaged in shared decision-making were more satisfied with the process of decision making than patients who reported making the decision on their own or that providers made the decision for them ( Dehlendorf *et al.*, 2017).

Patients appear to benefit from thorough education and moderate assistance in contraceptive decision-making. As such, the educator approach – with its emphasis on full information and assisting with choice through providing perspective – may be the best approach for driving patient satisfaction with their method of choice.

The arbiter approach, where the provider has the ultimate say in whether the procedure is offered, may generate reduced patient satisfaction. The arbiter approach is in some ways honoring the autonomy of the patient’s future self more than the autonomy of the patient’s current self as justified by avoiding harm. There is substantial merit to the need to minimize harm for sterilization procedures, particularly considering the legacy of forced sterilization in many countries around the world. However, it may be time to reexamine the tradeoff between addressing that legacy through cautious provision and addressing it through honoring patient autonomy. Forced sterilization was fundamentally a lack of autonomy, and truly addressing this legacy may best be served by prioritizing patient autonomy.

Clinical decision-making around sterilization has been studied more extensively for tubal ligations (female sterilization) than vasectomy. There are many parallel barriers to access for tubal ligations – young and childless women are commonly turned away when seeking tubal ligations over the risk of regret ( Lalonde, 2018; Richie, 2013). Notably, much of the current literature presumes regret is not factored into decision-making for men seeking vasectomies as it is for women seeking tubal ligations ( Mertes, 2017; Richie, 2013), though the findings from this study suggest otherwise. It is well-documented that regret is centrally factored into clinical decision-making for tubal ligation procedures ( Lalonde, 2018; Mertes, 2017; Richie, 2013; Taylor, 2020). Similarly, it is expected that age, number of children, and marital status are factored in as subsidiary characteristics as part of weighing a tubal ligation patient’s chance of regret ( Sobel & Gert, 1986). 

Case studies and commentaries have also reflected female sterilization providers striving to find a balance between autonomy and non-maleficence ( Goldrath & Smith, 2016). Presenting different perspectives on a case study for providers, Goldrath & Smith (2016) noted: “While it is the duty of the physician to “do no harm,” it is preferable to provide extensive counseling and allow the patient to decide, rather than to refuse unilaterally, which would be paternalistic….Ultimately, it is the responsibility of the health care provider to have a substantive discussion, fully inform the patient, provide alternative treatment options, and allow the patient to decide.” The counter perspective promoted the value of a six-month or one-year waiting period to manage both honoring autonomy and mitigating the risk of “tubal regret” harm.

Ultimately there are greater parallels to clinical decision-making between tubal ligation providers and vasectomy providers than much of the literature currently suggests. There are educator approaches and arbiter approaches among both tubal ligation and vasectomy providers. Yet in both instances, the arbiter approach has been challenged as paternalistic, unnecessarily regret cautious, and discriminatory. It is also worth noting that although providers appear to have similar approaches to counseling tubal ligations and vasectomies, tubal ligation procedures are significantly more invasive, riskier, and less reversible. It stands to reason that vasectomy providers can and should take a less cautious approach to non-maleficence in vasectomy provision, given the reduced level of risk compared to tubal ligations.

### Limitations & future research directions

There are several limitations of this study. One of the limitations of the study is the small sample size, tandem with broad geographic coverage. In some countries, only one provider was interviewed, which is not representative of all providers in the country. As an exploratory study, these results are not intended to be generalizable. Additionally, the sample may be subject to selection bias, as participants were recruited through convenience sampling. There was also a very low response rate to the initial screening email, which may have biased the sample. The eligibility requirement that providers have at least five years of experience may have also skewed the sample to an older demographic that may not include or demonstrate the perspectives of younger or more recently trained providers. The data collected through in-depth interviews are based on participant self-report and may not capture the full complexity or the actual decisions among providers. While we obtained informed consent as approved through an institutional ethics board, it is possible that some providers offered socially desirable responses, leading to potential data limitations. Nonetheless, this study is believed to be the first of its kind and offers important foundational considerations for the decision-making process among vasectomy providers in accessing vasectomy services. Further research can expand upon these concepts and seek to provide more regional specificity and generalizability, as well as produce localized recommendations. It may also be beneficial to replicate some of this research in the US in the wake of the Supreme Court’s decision in *Dobbs v Jackson* (2022) to ^1^ evaluate any impact of reduced abortion access on sterilization provision decision-making. Future research could also include examining how providers consider gender roles in evaluating patient candidacy.

### Implications

 The findings of this study suggest an opportunity for expanded training for vasectomy providers on procedural decision-making, pre-procedure consent, and clinical ethics, given the immense complexity of cases vasectomy providers encounter. Participant responses highlight the current gap in provider training, both in the procedure itself and in the consent and ethics surrounding sterilization procedures. At an individual level, it also invites vasectomy providers to examine and mitigate opportunities for bias to shape decision-making, including examining the role of evidence in their decision-making. Providers only reported evidence as driving their decision-making when it came to patients at high risk of regret, and some of the criteria they described being as high-risk are not consistently supported in the literature nor in the lived experience of those who provide reversals ( Masterson *et al.*, 2013; Najari *et al.*, 2021). There is a need to examine what kind of evidence is used and the way evidence is used to guide decision-making, as well as a need to practice reflexivity on how bias may shape decision-making. Future trainings of vasectomy providers should focus on evidence-based medicine, shared decision-making, and patient-centered care to ensure vasectomy provision that honors patient autonomy and rights.

 Improving access to existing contraceptive methods will be critically important to meeting Sustainable Development Goals on ensuring universal access to sexual and reproductive health and rights (Goal 5, Target 6). Within that, expanding access to non-permanent male methods of contraception will be essential, as will expanding access to existing methods including vasectomy. Future vasectomy training should also incorporate medical ethics training, including balancing the principles of autonomy and non-maleficence in decision-making, particularly as they relate to sterilization.

## Consent

Recorded oral informed consent was received for publication of the participants’ perspectives.

## Data Availability

The data that are the basis of this paper have not been uploaded to a publicly available database to protect participant confidentiality. Given participants are all members of a closed professional network, full transcript de-identification is not possible. The data are available upon IRB approval for secondary data analysis by contacting the corresponding author at alison.hoover@emory.edu.

## Footnotes

^1^ Dobbs v. Jackson (2022) 597 U.S. 215.
