# Supplementary material for: Vasectomy provider decision-making balancing autonomy and non-maleficence: qualitative interviews with providers
Source: Gates Open Res. 2024 Oct 7;7:132. Originally published 2023 Dec 6. [Version 2] doi: 10.12688/gatesopenres.15036.2 (PMC10861492; doi:10.12688/gatesopenres.15036.2)
Supplement: Peer review history

# Reviewer report: Reviewer response for version 2

**DOI:** 10.21956/gatesopenres.17673.r38229

**Published:** 2024-10-24

**Authors:** Trinity Zan

**Affiliations:** 1 Research Utilization, Global Health Population and Nutrition, FHI 360, Durham, NC, USA

I approve the revised manuscript.

Is the work clearly and accurately presented and does it cite the current literature?

Yes

If applicable, is the statistical analysis and its interpretation appropriate?

Not applicable

Are all the source data underlying the results available to ensure full reproducibility?

No source data required

Is the study design appropriate and is the work technically sound?

Yes

Are the conclusions drawn adequately supported by the results?

Yes

Are sufficient details of methods and analysis provided to allow replication by others?

Yes

Reviewer Expertise:

Family planning programs and policies, research utilization/research-to-practice/knowledge management, digital health.

I confirm that I have read this submission and believe that I have an appropriate level of expertise to confirm that it is of an acceptable scientific standard.

# Reviewer report: Reviewer response for version 2

**DOI:** 10.21956/gatesopenres.17673.r38230

**Published:** 2024-10-12

**Authors:** Jay Sandlow

**Affiliations:** 1 Urology, Medical College of Wisconsin, Milwaukee, WI, USA

The authors appeared to have addressed our major concerns.  We would recommend indexing

Is the work clearly and accurately presented and does it cite the current literature?

Partly

If applicable, is the statistical analysis and its interpretation appropriate?

Not applicable

Are all the source data underlying the results available to ensure full reproducibility?

Partly

Is the study design appropriate and is the work technically sound?

Partly

Are the conclusions drawn adequately supported by the results?

Partly

Are sufficient details of methods and analysis provided to allow replication by others?

Yes

Reviewer Expertise:

NA

I confirm that I have read this submission and believe that I have an appropriate level of expertise to confirm that it is of an acceptable scientific standard.

# Reviewer report: Reviewer response for version 1

**DOI:** 10.21956/gatesopenres.16370.r35839

**Published:** 2024-05-24

**Authors:** Philip S. Li, Joshua Halpern

**Affiliations:** 1 Department of Urology, Weill Cornell Medicine, New York City, NY, USA; 2 Northwestern University, Evanston, Illinois, USA

The authors present a qualitative analysis of provider decision-making in counselling patients regarding vasectomy. They used semi-structured interviews to understand provider perspectives on vasectomy, particularly in challenging cases with a higher potential risk of patient regret.

 The study is methodologically sound, with interesting thematic results from 15 providers encompassing diverse perspectives, backgrounds, and experiences. The clinical vignettes were well-chosen and represent some of the more challenging vasectomy consultations seen regularly in clinical practice.

 Certain important themes emerge from these long-form interviews. For example, the authors isolated and highlighted three foundational components crucial to patient candidacy in the counselling process: complete patient information, the ability to provide informed consent, and a clear and specific desire to proceed with vasectomy.

 Perhaps the most important dichotomy that emerges is the balance between patient autonomy and non-maleficence in patient counselling. The authors identify two general “types” of providers or two distinct approaches: "educators," who prioritise autonomy, and "arbiters," who prioritize non-maleficence.Overall, this is an interesting and comprehensive picture of the provider's perspective on vasectomy counselling from multiple vantage points. While no obvious or distinct barriers to care on the provider side are identified, the recognition that prioritization of non-maleficence may impact access to care is important and may inform future training and practice for those physicians performing vasectomy.

 Minor critiques include: a suggestion for the authors to provide the semi-structured interview guide as a supplement, enhancing transparency in their methodology. Additionally, this well-written manuscript could benefit from incorporating additional quotes from the interviews, adding depth and distinction to the presented findings. Overall, this study contributes valuable insights to the understanding of provider decision-making in vasectomy counselling, offering implications for both current practice and future professional development.

Is the work clearly and accurately presented and does it cite the current literature?

Yes

If applicable, is the statistical analysis and its interpretation appropriate?

Not applicable

Are all the source data underlying the results available to ensure full reproducibility?

No source data required

Is the study design appropriate and is the work technically sound?

Yes

Are the conclusions drawn adequately supported by the results?

Yes

Are sufficient details of methods and analysis provided to allow replication by others?

Yes

Reviewer Expertise:

Andrology, male infertility

We confirm that we have read this submission and believe that we have an appropriate level of expertise to confirm that it is of an acceptable scientific standard.

# Response

**Published:** 2024-10-07

**Authors:** Alison Hoover

**Affiliations:** Emory University, Atlanta, Georgia, USA

Minor critiques include: a suggestion for the authors to provide the semi-structured interview guide as a supplement, enhancing transparency in their methodology.

- Response: The semi-structured guide is now available via repository, with additional details on source and access in the “Data Availability Statement” section of the paper.

# Reviewer report: Reviewer response for version 1

**DOI:** 10.21956/gatesopenres.16370.r35705

**Published:** 2024-02-26

**Authors:** Trinity Zan

**Affiliations:** 1 Research Utilization, Global Health Population and Nutrition, FHI 360, Durham, NC, USA

This is a very well-written and thoughtful article. My comments seek to help further strengthen it.

 Bigger-picture/conceptual comments:

 I think it would be useful for the authors to provide more information about the selection of the terms "autonomy," "maleficence," and "cost-benefit analysis."  Specifically, it would be helpful to know if these terms have been previously used in other, similar research (public health, contraceptive, or other) and the extent to which they were pre-determined or arose after analysis of interviews. 

 The Figure 1 is helpful, and yet I found myself taking issue with the linear nature it represents. The results, as you share them, indicate a much more complex decision-making process, such that you might consider reworking the image. Specifically, it might be helpful to indicate that the pre-procedure counseling begins the process (perhaps taking the place of the foundational conditions), and that the three foundation conditions are actually considered throughout the process--so perhaps make those a backdrop/cross-cutting the patient characteristics and the provider context, and possible even the cost/benefit analysis. It seems the end product is the decision of how to weigh autonomy v. non-maleficence, so that could be represented as the "outcome." 

 In the implications section, I was a bit surprise to see you lead with the role of evidence, as this did not seem to come up much in the earlier results or discussion sections. Instead, I would focus on the fact that this type of counseling and decision-making is very complex, and therefore should be included and discussed directly in provider trainings; this in light of the fact that the interviewees all noted limited training on vasectomy in general, and counseling even more so. 

 Finally, the last paragraph--and more specifically, the second-to-last sentence--of implications section seems inappropriately opinionated and strong as a values-statement. The rest of the article is framed as being more exploratory and, in fact, you explicitly state under Conclusions that you will not attempt to evaluated the defensibility of the two paradigms. And yet, in the implications section, you argue for one over the other. I think the rest of the article works as-is, and you should consider rephrasing this statement and letting the audience make their own conclusions.

 Lastly, I like the discussion relating decision-making for female sterilization to vasectomy. I was expecting to see a bit more discussion about gender roles--perhaps whether/to what extent providers considered gender roles as part of their own cost-benefit analysis, for ex, whether they think that a couple making the decision has more/less potential for regret--but I didn't see anything. Just wondering if there is more that can be gleaned from the transcripts and considered related to gender roles.

 Smaller comments:

 In study design/participant recruitment, it would be helpful to more clearly define the criteria of being "an active vasectomy provider for a minimum of 5 years."  Does that mean at least one vasectomy per month? Some other definition?

 In the first paragraph under results, the last sentence (beginning "The interviewer reviewed notes...") and the following are repetitive of what is in the study design section and can be removed here.

Is the work clearly and accurately presented and does it cite the current literature?

Yes

If applicable, is the statistical analysis and its interpretation appropriate?

Not applicable

Are all the source data underlying the results available to ensure full reproducibility?

No source data required

Is the study design appropriate and is the work technically sound?

Yes

Are the conclusions drawn adequately supported by the results?

Yes

Are sufficient details of methods and analysis provided to allow replication by others?

Yes

Reviewer Expertise:

Family planning programs and policies, research utilization/research-to-practice/knowledge management, digital health.

I confirm that I have read this submission and believe that I have an appropriate level of expertise to confirm that it is of an acceptable scientific standard, however I have significant reservations, as outlined above.

# Response

**Published:** 2024-10-07

**Authors:** Alison Hoover

**Affiliations:** Emory University, Atlanta, Georgia, USA

**Reviewer comment: **I think it would be useful for the authors to provide more information about the selection of the terms "autonomy," "maleficence," and "cost-benefit analysis."  Specifically, it would be helpful to know if these terms have been previously used in other, similar research (public health, contraceptive, or other) and the extent to which they were pre-determined or arose after analysis of interviews. 

- **Author Response:** We agree with this suggestion. Context and a citation has been added to the “Cost-Benefit Analysis & Ethical Obligations” section. 

**Reviewer comment: **The Figure 1 is helpful, and yet I found myself taking issue with the linear nature it represents. The results, as you share them, indicate a much more complex decision-making process, such that you might consider reworking the image. Specifically, it might be helpful to indicate that the pre-procedure counseling begins the process (perhaps taking the place of the foundational conditions), and that the three foundation conditions are actually considered throughout the process--so perhaps make those a backdrop/cross-cutting the patient characteristics and the provider context, and possible even the cost/benefit analysis. It seems the end product is the decision of how to weigh autonomy v. non-maleficence, so that could be represented as the "outcome." 

- **Author Response:**Figure 1 has been revised. 

**Reviewer comment: **In the implications section, I was a bit surprise to see you lead with the role of evidence, as this did not seem to come up much in the earlier results or discussion sections. Instead, I would focus on the fact that this type of counseling and decision-making is very complex, and therefore should be included and discussed directly in provider trainings; this in light of the fact that the interviewees all noted limited training on vasectomy in general, and counseling even more so. 

- **Author Response:** This is a very valid consideration, and the “Implications” section has been revised accordingly.

**Reviewer comment: **Finally, the last paragraph--and more specifically, the second-to-last sentence--of implications section seems inappropriately opinionated and strong as a values-statement. The rest of the article is framed as being more exploratory and, in fact, you explicitly state under Conclusions that you will not attempt to evaluated the defensibility of the two paradigms. And yet, in the implications section, you argue for one over the other. I think the rest of the article works as-is, and you should consider rephrasing this statement and letting the audience make their own conclusions.

- **Author Response: **We appreciate this perspective and have revised the final paragraph to retain more neutrality.

**Reviewer comment: **Lastly, I like the discussion relating decision-making for female sterilization to vasectomy. I was expecting to see a bit more discussion about gender roles--perhaps whether/to what extent providers considered gender roles as part of their own cost-benefit analysis, for ex, whether they think that a couple making the decision has more/less potential for regret--but I didn't see anything. Just wondering if there is more that can be gleaned from the transcripts and considered related to gender roles.

- **Author Response:** This is an excellent consideration, and one that merits further consideration. Gender roles appear to have been considered more fully among providers in the “educator” group, which has now been noted in the “Discussion” section. The data were limited, and language has also been added to the “Limitations & Future Research Directions” section to encourage further research in this area.   

Smaller comments:

**Reviewer comment: **In study design/participant recruitment, it would be helpful to more clearly define the criteria of being "an active vasectomy provider for a minimum of 5 years."  Does that mean at least one vasectomy per month? Some other definition?

- **Author Response: **We did not use a standard definition of “active” provision, we just asked that people had been providing the services for a minimum of five years in the screening tool. We have modified the language slightly to more closely reflect the language in the screening tool.

**Reviewer comment: **In the first paragraph under results, the last sentence (beginning "The interviewer reviewed notes...") and the following are repetitive of what is in the study design section and can be removed here.

- **Author Response:** Agreed, this has been removed.

# Reviewer report: Reviewer response for version 1

**DOI:** 10.21956/gatesopenres.16370.r35843

**Published:** 2024-02-15

**Authors:** Jay Sandlow, Daniel Pelzman

**Affiliations:** 1 Urology, Medical College of Wisconsin, Milwaukee, WI, USA

The authors conducted a qualitative exploratory study examining the decision trees and ethical considerations of vasectomy providers to determine patient candidacy for sterilization. To do so, they interviewed 15 providers from the Vasectomy Network and analyzed the content of these interviews. They applied the interview themes to the ethical principles of autonomy and non-maleficence. The authors concluded that different vasectomy providers rely on different ethical principles to guide their patient counseling. They propose that improving vasectomy training and education may provide a better framework for counseling patients.

**Introduction**

 1. The introduction does a great job explaining the importance of increasing provider awareness/knowledge of vasectomy practices. However, redirecting the focus to the specific objectives of this paper would improve readability. For example, there is an entire paragraph devoted to comparing tubal ligation to vasectomy, yet the words “autonomy” and “non-maleficence” do not appear in the intro. Based on the conclusions, the major focuses of the paper are (1) how providers use autonomy and non-maleficence to counsel patients about vasectomy and (2) the merits of each approach. Providing some background about the ethical considerations you discuss later in the paper would strengthen this section.

**Methods**

 1. One of your inclusion criteria is being an active vasectomy provider for a minimum of 5 years to "ensure breadth of experience and...to draw on actual experience." However, in your Results you explain that providers use not only experience but also training, local laws, sociocultural norms, and peer influence to evaluate patient candidacy. Meanwhile, the median age of providers you surveyed was 65. Do you think that by not including younger (or more recently trained) providers you have introduced a source of bias to your results? In the limitations section, it would be helpful to mention this and propose how the generally older age of your study participants may have affected the results.

 2. Why do you think the screening e-mail had such a low response rate? It might be worth putting this in the limitation sections.

 3. You describe a complex method of data analysis involving MAXQDA20, inductive and deductive themes, codebook iteration, thematic analysis, etc. How did this contribute to the results? Did you use this data analysis to create the pre-procedure decision making flow in Figure 1?

 4. You then say that results were compared across age, gender, etc. What results were actually compared here and how were they compared? Should this comparison (even if negative or not statistically significant) go in the Results section?

**Results**

 1. The results are very detailed and do a good job summarizing the varied responses of the 15 providers. The “Patient characteristics” section might benefit from subsections similar to what you did with “Provider Landscape”.

**Discussion**

 1. In the first paragraph of the discussion, you explain that “prioritizing autonomy meant doing the procedure…and emphasizing non-maleficence…meant delaying or denying.” Do you mean this only for more complicated ethical cases? For routine vasectomy consultations, I do not think emphasizing non-maleficence would lead to a provider delaying or denying the case.

 2. You discuss the strengths and weaknesses of the educator and arbiter approaches. While the overall purpose of the section could be important, the examples provided are confusing and not supported by references within the paper. For example, you say that a weakness of the educator approach is creation of differential access to vasectomy reversals due to cost/availability. Is that really a direct consequence of patient education and autonomy? Certainly, the educator approach could be associated with more regret (although I am not aware of any literature to support this), but to say that it leads to differential access to care overlooks the complexities of healthcare inequity and access to infertility services worldwide.

 Similarly, what do you mean by “the arbiter approach is strong in that it avoids complications in patients that may come to regret the procedure”? Are you saying that the arbiter approach can reduce vasectomy complication rates? You also say that the arbiter approach “reduces costs and overall utilization of health care resources.” Do you have literature to back this up? I would argue that unwanted conception or female sterilization (for couples where the male was not deemed a vasectomy candidate) are far more expensive and resource-intensive than vasectomy.  Additionally, there is a more recent paper (Charles, et al.  Vasectomy Regret Among Childless Men. Urology. 2022) that specifically examines vasectomy regret.

 3. Implications section does a good job addressing the importance of this topic in the greater context of vasectomy training/education (or lack thereof) and relating this importance to UN’s Sustainable Development Goals.

**Overall**

1. The paper is well-written and addresses an important topic that unfortunately has very little surrounding literature. However, it is unclear whether the major focus is on the current landscape of vasectomy provider counseling or on the merits of autonomy and non-maleficence in the context of vasectomy. For example, the results are very specific and address many aspects of pre-vasectomy counseling – but only in the last section are ethical considerations addressed. The entire conclusion, though, focuses on autonomy and non-maleficence. It would be helpful reframe and condense the results in the context of ethical considerations so that the paper has one clear focus throughout. 

A few minor issues that would improve the clarity of the paper:

 1. Paragraph 1 of the introduction should be edited to say "...involves disrupting the flow of **sperm **(not semen) ..."

 2. Paragraph 2 of "Role of Pre-Procedure Counseling": I would suggest changing "...such a breaking up or divorcing" to "separating from the current partner."

 3. Paragraph 2 of "Foundational Characteristics": Can you expand upon the circumstances in which providers would proceed with vasectomy when a patient cannot consent?

 4. Paragraph 9 of "Patient Characteristics": You say that men on testosterone were not generally considered candidates for vasectomy unless they were making sperm. Was this a commonly held belief across providers? While it is true that T therapy can cause infertility, this is not a reliable form of contraception (certainly not more reliable than vasectomy). Additionally, sperm production can return shortly after stopping T. It would be unusual (and likely not indicative of current trends) for a panel of 15 vasectomy experts to generally exclude patients on T.

Is the work clearly and accurately presented and does it cite the current literature?

Partly

If applicable, is the statistical analysis and its interpretation appropriate?

Not applicable

Are all the source data underlying the results available to ensure full reproducibility?

Partly

Is the study design appropriate and is the work technically sound?

Partly

Are the conclusions drawn adequately supported by the results?

Partly

Are sufficient details of methods and analysis provided to allow replication by others?

Yes

Reviewer Expertise:

vasectomy, vasectomy reversal

We confirm that we have read this submission and believe that we have an appropriate level of expertise to confirm that it is of an acceptable scientific standard, however we have significant reservations, as outlined above.

# Response

**Published:** 2024-10-07

**Authors:** Alison Hoover

**Affiliations:** Emory University, Atlanta, Georgia, USA

Introduction

 1. The introduction does a great job explaining the importance of increasing provider awareness/knowledge of vasectomy practices. However, redirecting the focus to the specific objectives of this paper would improve readability. For example, there is an entire paragraph devoted to comparing tubal ligation to vasectomy, yet the words “autonomy” and “non-maleficence” do not appear in the intro. Based on the conclusions, the major focuses of the paper are (1) how providers use autonomy and non-maleficence to counsel patients about vasectomy and (2) the merits of each approach. Providing some background about the ethical considerations you discuss later in the paper would strengthen this section.

- Response: We agree the paper would benefit from additional context surrounding the ethical concepts used to underpin the conclusions of the study. Since they arose deductively during analysis and were not part of the rationale for the study, directly referenced in interviews or the interview guide, or touched on in each interview, we have elected to add context for the terminology and its use when the terms are first mentioned in the “Cost-Benefit Analysis & Ethical Obligations” section.

Methods

 1. One of your inclusion criteria is being an active vasectomy provider for a minimum of 5 years to "ensure breadth of experience and...to draw on actual experience." However, in your Results you explain that providers use not only experience but also training, local laws, sociocultural norms, and peer influence to evaluate patient candidacy. Meanwhile, the median age of providers you surveyed was 65. Do you think that by not including younger (or more recently trained) providers you have introduced a source of bias to your results? In the limitations section, it would be helpful to mention this and propose how the generally older age of your study participants may have affected the results.

- Response: Thank you for this important consideration of source of biases in the data, it has been added to the “Limitations & Future Research Directions” section.

2. Why do you think the screening e-mail had such a low response rate? It might be worth putting this in the limitation sections.

- Response: Also added to the “Limitations & Future Research Directions” section.

3. You describe a complex method of data analysis involving MAXQDA20, inductive and deductive themes, codebook iteration, thematic analysis, etc. How did this contribute to the results? Did you use this data analysis to create the pre-procedure decision making flow in Figure 1?

- Response: The data was used to create the pre-procedure decision-making flow in Figure 1, and this is now more clearly noted at the end of the “Methods” section

4. You then say that results were compared across age, gender, etc. What results were actually compared here and how were they compared? Should this comparison (even if negative or not statistically significant) go in the Results section?

- Response: The themes that emerged from the rich description of the factors involved in decision-making (all findings) were compared using thematic analysis. Analysis of significance was not conducted as part of this qualitative analysis. The “Methods” section has been modified to clarify this. 

Results

 1. The results are very detailed and do a good job summarizing the varied responses of the 15 providers. The “Patient characteristics” section might benefit from subsections similar to what you did with “Provider Landscape”.

- Response: The subsections have been added accordingly.

Discussion

 1. In the first paragraph of the discussion, you explain that “prioritizing autonomy meant doing the procedure…and emphasizing non-maleficence…meant delaying or denying.” Do you mean this only for more complicated ethical cases? For routine vasectomy consultations, I do not think emphasizing non-maleficence would lead to a provider delaying or denying the case.

- Response: In this study, complex cases were used to elicit decision-making processes among providers. Complexity lies on a spectrum, and the degree and extent to which providers adhere to the steps detailed in this paper varies. Some providers did express considering harm and delaying or denying a procedure even in less complex cases, such as a patient presenting with a pregnant partner (a vignette used in the interview guide of this study). Several providers, focusing on avoiding harm if the pregnancy is lost, described delaying the procedure under those circumstances. We have noted that the decision process described is particularly true in complex cases, but it is not necessarily untrue or irrelevant for more routine evaluations.

2. You discuss the strengths and weaknesses of the educator and arbiter approaches. While the overall purpose of the section could be important, the examples provided are confusing and not supported by references within the paper. For example, you say that a weakness of the educator approach is creation of differential access to vasectomy reversals due to cost/availability. Is that really a direct consequence of patient education and autonomy? Certainly, the educator approach could be associated with more regret (although I am not aware of any literature to support this), but to say that it leads to differential access to care overlooks the complexities of healthcare inequity and access to infertility services worldwide.

 Similarly, what do you mean by “the arbiter approach is strong in that it avoids complications in patients that may come to regret the procedure”? Are you saying that the arbiter approach can reduce vasectomy complication rates? You also say that the arbiter approach “reduces costs and overall utilization of health care resources.” Do you have literature to back this up? I would argue that unwanted conception or female sterilization (for couples where the male was not deemed a vasectomy candidate) are far more expensive and resource-intensive than vasectomy.  Additionally, there is a more recent paper (Charles, et al.  Vasectomy Regret Among Childless Men. Urology. 2022) that specifically examines vasectomy regret.

- Response: We appreciate these perspectives, and have added additional context and rephrasing, as well as the Charles citation, to the “Discussion” section accordingly.

A few minor issues that would improve the clarity of the paper:

 1. Paragraph 1 of the introduction should be edited to say "...involves disrupting the flow of sperm (not semen) ..."

- Response: Thank you for noting this, it has been changed accordingly.

2. Paragraph 2 of "Role of Pre-Procedure Counseling": I would suggest changing "...such a breaking up or divorcing" to "separating from the current partner."

- Response: This has also been changed accordingly.

3. Paragraph 2 of "Foundational Characteristics": Can you expand upon the circumstances in which providers would proceed with vasectomy when a patient cannot consent?

- Response: An example has been added to the “Foundational Characteristics” section.

4. Paragraph 9 of "Patient Characteristics": You say that men on testosterone were not generally considered candidates for vasectomy unless they were making sperm. Was this a commonly held belief across providers? While it is true that T therapy can cause infertility, this is not a reliable form of contraception (certainly not more reliable than vasectomy). Additionally, sperm production can return shortly after stopping T. It would be unusual (and likely not indicative of current trends) for a panel of 15 vasectomy experts to generally exclude patients on T.

- Response: We did not specifically query how providers would address patients on testosterone, but it did spontaneously come up in a couple of interviews. The providers that raised the subject did not mention intention to reconsider candidacy after stopping T. We have added clarification to that paragraph.

# Reviewer report: Reviewer response for version 1

**DOI:** 10.21956/gatesopenres.16370.r35706

**Published:** 2024-02-12

**Authors:** Heather Vahdat

**Affiliations:** 1 Male Contraceptive Initiative, Durham, USA

This article delves into the complicated nature of provider decision-making in the context of vasectomy provision. The authors have gathered a thorough and suggestive selection of influencing factors, despite the small sample size. 

 The manuscript is organized nicely, introducing the base decision-making criteria related to ensuring that a patient is certain about the procedure, is informed about the risks and realities of reversal, and consents to the procedure. These criteria align with the described current best practices for provision of patient-centered care and provide, as described by the authors, foundational decision-making criteria.

 It is when providers take into account the more subjective considerations of patient age, number of children, etc., that the decision-making process disassociates based on subjective interpretations of patient autonomy, non-maleficence, and the appropriate balance of the two concepts. Some providers skew more favorably towards patient autonomy dubbed "educators" by the authors, while others focus more on influencing patient decision making based on the provider's interpretation that non-maleficence is of greater concern than autonomy; the “arbiters”. 

 These findings highlight key areas to be addressed to improve quality and equity of care for men and their partners. First, it appears that clinicians may tend to only weigh the risk of vasectomy complications in the context of the presenting patient. This is a flawed calculation as the comparison is not between vasectomy complications and a patient not being vasectomized. Rather, the calculation should be based on the complication rates of vasectomy compared to those of tubal ligation and compared to the long-term financial, social, and emotional complications associated with an unintended pregnancy.  Second, the clinicians in this analysis tended to dismiss the patient’s partner's contraceptive and reproductive health journey (generally speaking) in their decision making. The exception being in cases of older patients with partners nearing menopause. In these cases, patients often receive additional counseling as providers weigh the minimal, but non-zero risks of vasectomy complications more heavily than a potential unintended pregnancy in this later stage of reproduction.

 The manuscript provides a considerable level of analysis and does an excellent job of deciphering and presenting the nuances of clinician's perceived roles in the context of providing permanent contraception. It also raises some very interesting questions about how clinicians view risk and benefit in the context of men’s involvement in preventing unintended pregnancy. These are critical insights for the field, particularly as more male contraceptive methods enter the market in the coming years. We must evolve from a purely dichotomous mindset of contraceptive counseling focused on female versus male contraceptives and expand to include a more inclusive, dyadic view of shared risk of unintended pregnancy. (see Campella, et al. 2020) (Ref 1). It is particularly concerning in the context of the arbiter archetype where denying a client access to vasectomy introduces risk of unintended pregnancy not only to their immediate client, but to a female partner who, in many environments, has even less autonomy for reproductive decision-making. As more male methods are rolled out, opportunities to refine and reinforce guidelines and best practices will be presented and should be harnessed.

 I have no specific feedback for the authors but do have one question for their consideration. My recommendation to approve  this manuscript is not contingent upon this question being answered.

 - Is it possible to speak to the actual rates of legal action taken due to vasectomy regret given the role that this concern plays in clinician decision-making? This may not be possible given the variation in countries where responding clinicians practice, etc. However, if it is possible to demonstrate the actual pervasiveness of this concern, that may be an interesting addition to the manuscript.

Is the work clearly and accurately presented and does it cite the current literature?

Yes

If applicable, is the statistical analysis and its interpretation appropriate?

Not applicable

Are all the source data underlying the results available to ensure full reproducibility?

Yes

Is the study design appropriate and is the work technically sound?

Yes

Are the conclusions drawn adequately supported by the results?

Yes

Are sufficient details of methods and analysis provided to allow replication by others?

Yes

Reviewer Expertise:

Reproductive health; contraception; public health

I confirm that I have read this submission and believe that I have an appropriate level of expertise to confirm that it is of an acceptable scientific standard.

## References

[1] : "Shared risk": Reframing risk analysis in the ethics of novel male contraceptives. *Contraception* .2020;102(2) : 10.1016/j.contraception.2020.05.014 67-69 10.1016/j.contraception.2020.05.014 32474061 PMC8168714

# Response

**Published:** 2024-10-07

**Authors:** Alison Hoover

**Affiliations:** Emory University, Atlanta, Georgia, USA

Is it possible to speak to the actual rates of legal action taken due to vasectomy regret given the role that this concern plays in clinician decision-making? This may not be possible given the variation in countries where responding clinicians practice, etc. However, if it is possible to demonstrate the actual pervasiveness of this concern, that may be an interesting addition to the manuscript.

- Response: We appreciate this idea, and have added additional detail to the “Role of Pre-Procedural Counseling” section.

# Reviewer report: Reviewer response for version 1

**DOI:** 10.21956/gatesopenres.16370.r35846

**Published:** 2024-02-02

**Authors:** Logan Nickels

**Affiliations:** 1 The Lundquist Institute for Biomedical Innovation at Harbor-UCLA Medical Center, Torrance, California, USA

This writing covers a study that interviews fifteen vasectomy providers about challenging cases and their determination of patient eligibility in order to describe the decision-making rationale of the providers. Using observations and quotes, the authors describe a bifurcated school of provider thought on patient decision-making wherein providers either act as "educators" or "arbiters" in evaluating the candidacy of a patient for vasectomy. Using clinical best practices, the authors go on to advocate for the "educator" approach, emphasizing patient autonomy.

 Positive comments are that it contains meaningful and descriptive analysis, thoughtful comparisons to existing literature, and considers reproductive healthcare bias for an understudied population - men. Additionally, the limitations of the study - primarily it's size, recruitment, and geographic focus, are well recognized and put into appropriate context.

 Criticisms are few, but specific comments and questions follow:

 Are there more references to support the claim in the first paragraph that vasectomy contributes to driving equitable gender norms? Could it not be the opposite, that men with equitable gender norms are more likely to use contraceptive methods? See citation 1.

 In the final sentence of the introduction - how will addressing supply gaps influence demand gaps as they have been described in the introduction? Their relationship and influence on each other is not clear to this reviewer. Moreover, if it has been suggested that supply is not the main issue behind low vasectomy uptake, does that lessen the importance of this writing? 

 Did any of the providers comment on how often they see difficult cases, or if the vignettes were commonplace in any way?

 Genuine question - In the Methods, should "google group" be capitalized? 

 How many of the providers offered reversals, and how many did not? Were there any differences in how those that provided reversals treated certain vignettes, or viewed the risk of regret versus those that did not?

 Was the repeal of Roe mentioned by any of the US providers as motivation, or potentially influential on their decision-making?

 More context, information, and citations regarding the history of forced sterilization in specific countries would be helpful for this reviewer.

 In the final paragraph - why call out non-hormonal methods of male contraception in particular?

 Regarding the reviewer question:

**Are all the source data underlying the results available to ensure full reproducibility?**

 The authors have made the data available on request, given the challenges with de-identification.

Is the work clearly and accurately presented and does it cite the current literature?

Yes

If applicable, is the statistical analysis and its interpretation appropriate?

Not applicable

Are all the source data underlying the results available to ensure full reproducibility?

Partly

Is the study design appropriate and is the work technically sound?

Yes

Are the conclusions drawn adequately supported by the results?

Yes

Are sufficient details of methods and analysis provided to allow replication by others?

Yes

Reviewer Expertise:

Male reproduction, contraception

I confirm that I have read this submission and believe that I have an appropriate level of expertise to confirm that it is of an acceptable scientific standard.

## References

[1] : Men's willingness to use novel male contraception is linked to gender-equitable attitudes: Results from an exploratory online survey. *Contraception* .2023;123: 10.1016/j.contraception.2023.110001 110001 10.1016/j.contraception.2023.110001 36924819

# Response

**Published:** 2024-08-03

**Authors:** Alison Hoover

**Affiliations:** Emory University, Atlanta, Georgia, USA

We thank the reviewers for their time and thoughtful feedback, which contributed to an improved and strengthened manuscript. Our responses to their suggested revisions are below.

**Reviewer 1**

 Are there more references to support the claim in the first paragraph that vasectomy contributes to driving equitable gender norms? Could it not be the opposite, that men with equitable gender norms are more likely to use contraceptive methods? See citation 1.

- Response: We thank the reviewer for introducing this new publication, and have revised the final sentence in the “Introduction” section accordingly and added the suggested citation alongside another article.

In the final sentence of the introduction - how will addressing supply gaps influence demand gaps as they have been described in the introduction? Their relationship and influence on each other is not clear to this reviewer. Moreover, if it has been suggested that supply is not the main issue behind low vasectomy uptake, does that lessen the importance of this writing? 

- Response: The reviewer raised an important consideration about the need for additional evidence regarding the link between supply and demand gaps in vasectomy. This link may best be explored in future write ups. The final paragraph of the “Introduction” section has been revised accordingly. 

Did any of the providers comment on how often they see difficult cases, or if the vignettes were commonplace in any way?

- Response: The vignettes were chosen during study design given their frequency of mention in the Google Group listserv, and are therefore considered to have been confronted by all providers with at least five years of experience (providing vasectomies for at least five years was part of the inclusion criteria for the study). No provider indicated that they had not seen a case included in the vignettes, but commonality was not measured in this study.

Genuine question - In the Methods, should "google group" be capitalized? 

- Response: Based on published preferences from Google to have it capitalized, I have capitalized “Google Group” in all mentions accordingly.

How many of the providers offered reversals, and how many did not? Were there any differences in how those that provided reversals treated certain vignettes, or viewed the risk of regret versus those that did not?

- Response: We did not specifically collect data on if providers offered reversals. Some providers volunteered that information during their interviews – three confirmed they provide vasectomy reversals, four confirmed they do not – but we cannot conclude how many of the remaining providers do or do not provide reversals. As noted in the final paragraph of the “Role of Pre-Procedure Counseling” section, the providers who offer reversals and vasectomies did report that their experience with reversals shaped their counseling practice and their decision-making with vasectomy patients. For the sake of this study, as noted in the third paragraph of the “Conclusions” section, whether or not a provider who was known to offer reversals did not appear to meaningfully impact whether they were more likely to prioritize autonomy or non-maleficence.

Was the repeal of Roe mentioned by any of the US providers as motivation, or potentially influential on their decision-making?

- Response: This is an interesting consideration – however this research took place in December 2020 prior to the repeal of Roe v Wade through the Dobbs decision. It would be interesting to interview providers about their decision-making since the Dobbs decision, and has been added to the “Limitations & Future Research Directions” section.

More context, information, and citations regarding the history of forced sterilization in specific countries would be helpful for this reviewer.

- Response: We have added additional context and a new citation to the “Laws and Guidelines” subsection with one of the first mentions of forced sterilization.

In the final paragraph - why call out non-hormonal methods of male contraception in particular?

- Response: Thank you for noting that – that should have said non-permanent, not non-hormonal. We have adjusted it accordingly. 
